# Combining Effect and Process Evaluation on European Preschool Children’s Snacking Behavior in a Kindergarten-Based, Family-Involved Cluster Randomized Controlled Trial: The ToyBox Study

**DOI:** 10.3390/ijerph17197312

**Published:** 2020-10-07

**Authors:** Marieke De Craemer, Vera Verbestel, Maïté Verloigne, Odysseas Androutsos, Luis Moreno, Violeta Iotova, Berthold Koletzko, Piotr Socha, Yannis Manios, Greet Cardon

**Affiliations:** 1 Department of Rehabilitation Sciences, Ghent University, 9000 Ghent, Belgium; vera.verbestel@ugent.be; 2 Research Foundation Flanders, 1000 Brussels, Belgium; maite.verloigne@ugent.be; 3 Department of Public Health and Primary Care, Ghent University, 9000 Ghent, Belgium; 4 Department of Nutrition and Dietetics, School of Physical Education, University of Thessaly, Sport Science and Dietetics, 421 00 Trikala, Greece; oandroutsos@uth.gr; 5 Department of Physiatry and Nursing, GENUD (Growth, Exercise, Drinking Behaviour and Development), University of Zaragoza, 50001 Zaragoza, Spain; lmoreno@unizar.es; 6 Department of Pediatrics, Medical University Varna, 9002 Varna, Bulgaria; iotova_v@yahoo.com; 7 Dr. von Hauner Children’s Hospital, University of Munich Medical Centre, 80337 Munich, Germany; office.koletzko@med.uni-muenchen.de; 8 Children’s Memorial Institute, 04-730 Warsaw, Poland; p.socha@czd.pl; 9 Department of Nutrition and Dietetics, School of Health Sciences & Education, Harokopio University, 17778 Athens, Greece; manios@hua.gr; 10 Department of Movement and Sports Sciences, Ghent University, 9000 Ghent, Belgium; greet.cardon@ugent.be

**Keywords:** intervention, effect evaluation, process evaluation, snacking behavior, preschoolers

## Abstract

This study aimed at (1) studying the effect of the standardized ToyBox intervention on European preschoolers’ snacking behavior, and (2) studying whether a higher process evaluation score from teachers and parents/caregivers was associated with a more positive result for preschoolers’ snack intake. A sample of 4970 preschoolers (51.4% boys, 4.74 ± 0.44 years) from six European countries provided information on snack intake with the use of a Food Frequency Questionnaire. To investigate the effect of the intervention, multilevel repeated measures analyses were executed for the total sample and the six country-specific samples. Furthermore, questionnaires to measure process evaluation were used to compute a total process evaluation score for teachers and parents/caregivers. No significant intervention effects on preschoolers’ snack intake were found (all *p* > 0.003). In general, no different effects of the intervention on snack intake were found according to kindergarten teachers’ and parents’/caregivers’ process evaluation scores. The lack of effects could be due to limited intervention duration and dose. To induce larger effects on preschoolers’ snack intake, a less standardized intervention which is more tailored to the local needs might be needed.

## 1. Introduction

A dramatic increase in childhood overweight and obesity has occurred in recent decades [1,2]. The development of overweight and obesity is a multifactorial health problem. Next to unmodifiable factors (e.g., genetic factors), modifiable lifestyle factors also have a large influence. The co-existence and interaction of energy balance-related behaviors (i.e., dietary intake, sedentary behavior, and physical activity) determine whether or not a positive energy balance is experienced [3,4,5]. 

A review looking at the association between snack intake and weight status in children and adolescents included five studies that reported on the relationship between preschoolers’ snack consumption and their weight status, of which four studies reported a positive association and one study reported an inverse relationship between preschoolers’ snack consumption (frequency of snack intake/day) and being overweight [6]. This could also be due to the fact that most studies did not distinguish between healthy or unhealthy snacks. The authors mentioned that targeting snack consumption in preschool children might be a promising area of research, since the consumption of snacks in addition to regular meals also increases energy intake and has the possibility to contribute to an energy imbalance in which more energy is consumed than expended. However, the authors also stated that no definite causal conclusions could be drawn between preschoolers’ snack consumption and overweight [6]. It is, however, plausible that snacking behavior is linked to the development of overweight and obesity in preschoolers. Therefore, the ToyBox study aimed to develop, implement, and evaluate a European kindergarten-based intervention with family involvement to prevent overweight and obesity in four- to six-year-old children from six countries (i.e., Belgium, Bulgaria, Germany, Greece, Poland, and Spain) [7,8]. The intervention focused on preschoolers’ healthy snacking behavior, sedentary behavior, physical activity, and water consumption. 

Investigating the effectiveness of interventions is important in order to understand the impact on different study outcomes. However, putting the effects of interventions into perspective by linking them to the process evaluation of the intervention ensures a better understanding of the potential effects of health promotion interventions [9]. For example, a lack of intervention effects might be due to the fact that some participants hardly received any intervention, which makes it important to take the received dose into account in the evaluation. To include process evaluation, a framework should be used to guide the process. The model of Saunders et al. (2005) was adapted in the ToyBox study and is described in detail elsewhere [10,11]. 

In the literature, some intervention studies focusing on energy-balance related behaviors already combined effect and process evaluation in intervention studies [12,13,14,15,16]. For example, a study in Belgian preschool and primary school children within the IDEFICS intervention (Identification and prevention of Dietary- and lifestyle-induced health EFfects In Children and infantS) showed that a higher process evaluation score was associated with favorable effects on children’s physical activity levels and their time spent sedentary [12]. Another study, although in adolescents, showed positive effects on the intake of fruit and vegetables when a higher process evaluation score was found [13]. Within the ToyBox study, the combination of process and effect evaluation has already been investigated for physical activity, sedentary behavior, and beverage consumption [17,18,19], but the results for snacking behavior have remained unstudied until now. 

Consequently, the aim of the current study was two-fold. First, we aimed to study the effectiveness of the ToyBox intervention on preschoolers’ healthy snacking behavior in the total sample and in the six country-specific samples. Second, we aimed to study whether a higher-level process evaluation score on the module related to healthy snacking behavior in the ToyBox intervention was related to more positive effects on preschoolers’ healthy snacking intake in the total sample. 

## 2. Materials and Methods 

### 2.1. Study Protocol

The ToyBox study was a large European project, from which partial results already have been published previously for physical activity [17], sedentary behavior [18], and beverage consumption [19]. Six European countries (i.e., Belgium, Bulgaria, Germany, Greece, Poland, and Spain) conducted a kindergarten-based, family-involved intervention with a cluster randomized controlled design targeting preschool children between four and six years old. Preschool children and their parents/caregivers were recruited at kindergartens, day-care centers, or preschool settings, depending on the country regulations and legislation. In Germany, Bulgaria, Spain, and Poland, children/families were recruited from kindergartens; in Greece, they were recruited from kindergartens and day-care centers; and in Belgium, they were recruited from preschool settings. In order to avoid confusion for the reader, all these settings (kindergartens, day-care centers, preschool settings) will be referred to as “kindergartens” in this paper. 

Kindergartens were recruited from West and East Flanders (Belgium), Varna (Bulgaria), Bavaria (Germany), Attica (Greece), Mazowiecki (Poland), and Zaragoza (Spain) and were selected from neighborhoods of different socio-economic statuses (SESs). All the cities and municipalities within the aforementioned provinces in each country were listed and ranked according to their SES (based on years of education or annual income at the level of the city or municipality, depending on the data availability within the respective city or municipality). The list was then split into tertiles, resulting in a group of cities and municipalities with a low SES, medium SES, and high SES. From each tertile in each country, five cities or municipalities were randomly selected (approximately five cities or municipalities for low SES, five for medium SES, and five for high SES). Eventually, 1003 kindergartens were randomly selected from the randomly chosen municipalities across the six European countries. However, the lowest 20% of kindergartens with the smallest number of children were excluded (e.g., if 10 kindergartens were present in one municipality, the two kindergartens with the least number of children were not contacted). The kindergarten staff was informed about the ToyBox study by a personal visit. 

The sample of the ToyBox study was previously described and published by Manios et al. (2014) [7], and the flow of kindergartens and participants through the study were also published in the same manuscript [7]. Across the six countries, 309 kindergartens (30.8%) decided to contribute in the ToyBox study. All the preschool children born in 2007 and 2008 (*n* = 16,798; age at baseline was 3.5–5.5 years old) were invited to participate in the study. Preschool children received an information letter in which the aim of the ToyBox study was clarified to the parents/caregivers. In total, 7056 parents/caregivers (42.0%) provided consent for their child to participate in the study. 

After the recruitment of kindergartens and to avoid contamination between kindergartens in the same municipality, kindergartens’ municipalities were randomly assigned to the intervention or control group (2:1). The project coordinator (Greece) used a command in Excel for randomization, which means that this happened in an automatic and electronic way. Kindergartens that were assigned to the intervention group received the intervention material, which could be used during school year 2012–2013. Kindergartens allocated to the control condition received the intervention material one year later and could continue with their normal curriculum. 

At the onset of the ToyBox study, power analyses were conducted with the use of specific software (http://www.statisticalsolutions.net). Power analyses were based on a previous preschool-based intervention study [20]. The main outcome used in the power analyses was preschool children’s body mass index (BMI)—namely, differences in BMI. Based on the recent literature [21], a baseline value for preschool children’s BMI of 16.35 kg/m^2^, an expected follow-up value of 16.17 kg/m^2^, a standard deviation of 1.73, an α-value of 0.05, and a power of 0.80 were used. This resulted in a sample of at least 726 preschool children which should be achieved. Therefore, the goal was to end up with a minimum sample of 800 preschoolers per intervention country with complete data at baseline and follow-up. To take possible drop-out into account, every intervention country recruited a minimum sample of at least 1100 preschoolers [7].

Baseline assessments were conducted on schooldays from May until June 2012 before the start of the intervention. Follow-up measurements took place one year later, from May until June 2013.

The ToyBox study was approved by the Ethical Committees in all European countries, in line with national regulations. The ToyBox study is registered with the clinical trials registry clinicaltrials.gov, ID: NCT02116296.

### 2.2. The Healthy Snacking Module

The Intervention Mapping protocol [22] was used to plan and develop the standardized ToyBox intervention and was similar in all countries. The Intervention Mapping protocol provides a systematic, stepwise framework for planning, implementing, and evaluating an intervention based on existing scientific literature, theories, and research data [22]. The intervention consisted of modules on sedentary behavior, physical activity, and water consumption, as well as a healthy snacking module, which will be the main outcome of this study. More details on the development of the snacking module using the Intervention Mapping protocol are described elsewhere (paper submitted).

**Figure 1:**
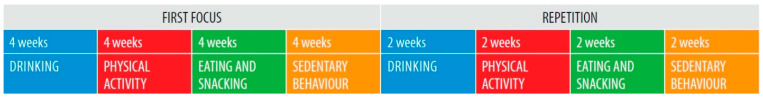
Flow of the 24-week ToyBox intervention.

The ToyBox intervention consisted of 24 weeks (Figure 1). The healthy snacking module of the intervention was conducted during the first focus (i.e., weeks 9 until 12), and was repeated for two weeks (i.e., weeks 21 and 22). In addition, throughout the whole intervention some healthy snacking components were also implemented. The ToyBox intervention was conducted by the kindergarten teachers, who followed (prior to the intervention) two teacher training sessions of one hour each with the researchers [23]. The aim of the sessions was to reply to kindergarten teachers’ questions and to clarify the aims of the ToyBox study and the material that would be used. During the second teacher training, kindergarten teachers received the “ToyBox”—i.e., a trunk holding material for the classroom environment (i.e., a teachers’ guide, classroom activity guides (one for each of the behaviors) and a hand puppet of a kangaroo) and the home environment (i.e., newsletters, tip cards, and posters for the parents/caregivers). The classroom activity guide for healthy snacking comprised three parts, with specific activities for each part—namely, (1) changes in the classroom environment (i.e., the use of the magical snack plate in kindergarten provides a variety of healthy snacks for the children, or the children can bring them from home and put them on the plate), (2) conducting the requested healthy behavior (i.e., eating healthy snacks together), and (3) classroom activities (i.e., kangaroo stories, sensory perception games, experiments, and excursions). Teachers were requested to spend at least 60 min on a weekly basis employing the materials and conducting the intervention in the classroom environment. The changes that were made in the classroom were implemented and preserved throughout the rest of the school year. Focus groups [24] were conducted before the development of the intervention and were used as a basis for the one hour per week guideline. Due to their busy week schedule, the kindergarten teachers clearly stated that they only could dedicate restricted time to the implementation and that ready-to-use materials had to be included [24]. Therefore, a minimum of 60 min per week was chosen as threshold, although devoting more time per week was recommended and encouraged. 

Preschool children received two newsletters, two tip cards, and one poster, with the aim to engage the parents/caregivers. The newsletters and tip cards contained tips and strategies to promote the consumption of healthy snacks in preschool children (e.g., making the fruit and vegetables available every day, not eating in front of television). All the materials are available on the website of the ToyBox study [25]. 

### 2.3. Procedure

Measurements were conducted according to standardized protocols. The procedure of data collection, data deposition, and data reporting was standardized and harmonized within the ToyBox study [26]. The researchers visited kindergartens and provided preschool children with two parental questionnaires (Primary Caregiver’s Questionnaire and Food Frequency Questionnaire (FFQ)) in a closed envelope to take home for completion by one of the parents/caregivers [27,28]. Afterwards, the questionnaires were collected when the researchers revisited the different kindergartens. 

### 2.4. Measurement of Snacks

The intake of snacks was measured by the FFQ, a valid and reliable questionnaire to measure preschool children’s food intake [28]. A standard, universally accepted definition for snacking is currently still lacking [29]. The definition used in the current study is a small portion of food eaten in between regular meals. Healthy snacks were defined as small portions of food without the addition of sugar or a high fat content. Unhealthy snacks were defined as small portions of food with added sugar or a high fat content.

Intakes of yoghurt (i.e., plain yoghurt), sugared or aromatized yoghurt, cheese, fresh fruit, raw vegetables, milk-based desserts (e.g., chocolate mousse, ice cream, custard), chocolate and candy bars (e.g., plain chocolate bars, chocolate candy bars), sugar-based desserts (e.g., hard candies, jelly beans, lollipops), cakes, biscuits, and salty snacks (e.g., potato chips) were each assessed with two food-frequency questions. First, on a six-point scale ranging from “never or less than once a month” to “every day”, parents/caregivers were asked on how many days per week their child consumed a snack. Subsequently, they were asked to indicate how much their child ate on days they consumed the snack. Parents/caregivers had to tick the average amount per day for each of the snacking categories. The possible answer categories are depicted in Supplementary file 1.

The mean intakes in g/day were calculated from the FFQ by the multiplication of the number of days/week and amount per day in g (using the midpoint method), divided by seven (total of number of days in a full week). This reflected the mean daily intake of each food category. Afterwards, the mean daily intakes of healthy and unhealthy snacks were calculated by adding up different categories that reflected either healthy or unhealthy snacks. For unhealthy snacks, these food categories were sugared or aromatized yoghurt, milk-based desserts, chocolate and candy bars, sugar-based desserts, cakes, biscuits, and salty snacks. For healthy snacks, the following categories were added up: plain yoghurt, cheese, fresh fruit, and raw vegetables. 

### 2.5. Process Evaluation

The process evaluation protocol in this study was guided by the specific model by Saunders et al. (2005) by assessing some key process evaluation elements [10,11]. Several key elements to conduct process evaluation were described in the process evaluation model developed specifically for the ToyBox study: (1) reach (level of contribution in the study); (2) fidelity (quality of implementation); (3) dose delivered (the quantity of the intervention that was implemented by the implementers); (4) dose received—exposure (the level of active contribution and being open to or using the materials and resources); (5) dose received—satisfaction (the level of gratification of the implementer and the target group regarding the intervention); and (6) context (in which the ToyBox intervention was delivered) [10]. Questions were developed to quantify the intervention process and satisfaction. “Fidelity”, “Dose delivered”, and “Dose received–satisfaction” were the process evaluation components that were assessed in the monthly logbooks [10,11]. 

Table 1 depicts the different questions per process evaluation component that were taken from the logbooks with a clarification about the configuration of the score. Teachers completed two logbooks, one during the first period and one during the second period [30]. Both logbooks were completed through email or with the use of phone calls. Questions from the logbooks that had answer categories on a 5-point scale were recoded into 0 (below the mean) or 1 (equal to or higher than the mean). For each intervention kindergarten, the scores on all the questions were added up to compute the total process evaluation score. A higher level of implementation at kindergartens is represented by a higher process evaluation score. The process evaluation score ranged between 0 and 24. After calculating the teachers’ process evaluation score, the kindergartens were distributed and recoded into tertiles (low score = 1; medium score = 2; high score = 3). In addition, the control group was also added (recoded into 0) to study the difference in effect on preschoolers’ snacking behavior according to the process evaluation (three categories: 1, 2, 3).

### 2.6. Process Evaluation: Parents/Caregivers

Parents/caregivers also filled in a process evaluation questionnaire that was added to the effect questionnaire at follow-up to obtain process evaluation measures of the snacking module of the intervention, which enabled us to assess the key process evaluation elements. Again, the model of Saunders et al. (2005) was used as a basis for the questions [10,11]. “Dose delivered”, “Dose received—exposure”, and “Dose received—satisfaction” were the process evaluation components that were assessed in the questionnaire for parents/caregivers [10,11]. 

Table 2 depicts the different questions per component that were used from the questionnaire with an elaboration about the score. Questions that had answer categories on a 5-point scale were recoded into 0 (lower than the mean) and 1 (equal to or higher than the mean). A total process evaluation score was calculated by summing all the scores. Again, a higher level of intervention implementation was represented by a higher process evaluation score. Parents’/caregivers’ process evaluation scores ranged from 0 to 17. After calculating the parents’/caregivers’ process evaluation score, the preschool children were distributed and recoded into tertiles (low score = 1; medium score = 2; high score = 3). In addition, the control group was again added as an additional group (recoded into 0) to study the difference in effect on preschool children’s snacking behavior according to the parents’/caregivers’ process evaluation score (three categories: 1, 2, 3).

### 2.7. Statistical Analyses

The characteristics (age, sex) of the sample were defined by computing descriptive statistics and were described as frequencies (%) or means and standard deviations. To check whether differences in the intake of healthy and unhealthy snacks between intervention and control group were already present, independent sample *t*-tests were performed using SPSS version 25 (IBM Corporation, Chicago, IL, USA).

MLwiN 2.31 (Centre for Multilevel Modelling, University of Bristol, Bristol, UK) was used to perform multilevel repeated measures analyses to evaluate the effect of the intervention on the different food categories, as well as on healthy and unhealthy snacks. To take the clustering of two measurements into account (baseline and follow-up) for preschool children in classrooms in kindergartens in six different countries, multilevel modelling was used (five levels). In the results, two values will be reported: (1) the value for the time effect can be elucidated as the size of change in the outcome from follow-up to baseline for the reference category (i.e., control group); (2) the ß-value for “time*condition” is the size of the intervention effect of the outcome, which describes the difference between the mean change in the intervention group and the control group. 

To take the effect of the intervention process on preschool children’s snacking behavior into account, multilevel repeated measures analyses (five levels) were again performed (“time*process evaluation score”). All the analyses were corrected for preschool children’s sex and age. Completer analysis was carried out to handle missing data, which means that only preschoolers with valid data at both baseline and follow-up were included in the analyses. For all the analyses, Bonferroni corrections were performed due to the high number of dependent variables (*n* = 13), inducing the fact that statistical significance was set at *p* < 0.003.

## 3. Results

In total, information on food intake was provided for 4970 children (51.4% boys, age at baseline 4.74 ± 0.44 years), together with their parents/caregivers and teachers providing information on the process of the intervention. At baseline, preschool children had an average intake of 59.63 g (±48.54) and 231.45 g (±135.68) of unhealthy and healthy snacks per day, respectively. At follow-up, they had an average intake of 71.86g (±53.62) and 227.59 (±131.74) of unhealthy and healthy snacks per day, respectively. The intervention and control group did not have a different intake of unhealthy snacks (*t* = 0.50, *p* = 0.62) and healthy ones (*t* = 1.33, *p* = 0.19) at baseline. Descriptive analyses showed that there was no significant difference between the intervention and control group regarding sex (chi^2^ = 0.48, *p* = 0.49), age (*t* = 1.15, *p* = 0.25), and the intake of unhealthy snacks at baseline (*t* = 0.50, *p* = 0.62). The CONsolidated Standards Of Reporting Trials (CONSORT) checklist was added in Supplementary file 2.

### 3.1. Intervention Effects: Total Sample

Table 3 shows the results for the total sample. There were no significant intervention effects for the different included food items (i.e., yoghurt, cheese, fresh fruit, vegetables, aromatized yoghurt, chocolate, milk-based desserts, cake, biscuit, sugar-based desserts, salty snacks, healthy snacks, and unhealthy snacks). 

### 3.2. Country-Specific Intervention Effects

Country-specific results are shown in Table 4. Only two significant intervention effects were found—one in the Bulgarian sample and one in the Spanish sample. An inverse intervention effect (*p* = 0.03) was found for sugar-based desserts with Bulgarian preschoolers from the intervention group having an increase (+0.87 g/day) from baseline to follow-up compared to a decrease in the control group (−0.16 g/day). In Spain, the preschoolers from the intervention group experienced a larger decrease in milk-based desserts (−6.07 g/day) from baseline to follow-up (*p* = 0.009) compared to children from the control group (−0.83 g/day). 

### 3.3. Association Teachers’ Process Evaluation Score and the Effects on Preschool Children’s Snacking Behavior (Total Sample)

For 3255 children out of 4970, a teachers’ process evaluation score could be calculated. This demonstrates that not all teachers completed the logbooks. In total, 460 teachers contributed to the intervention. However, 21.7% of teachers (*n* = 100) did not complete the first logbook for snacking behavior, and 30.9% (*n* = 142) of teachers did not fill in the second logbook for snacking behavior. The mean teachers’ process evaluation score was 14.58 (±3.70) on a total score of 24 (range: 3–23). The division of the kindergartens into tertiles based on the teachers’ process evaluation score was as follows: (1) low score (range 3.00–12.60; *n_children_* = 1048; *n_kindergartens_* = 43), (2) medium score (range 13.00–16.33; *n_children_* = 1115; *n_kindergartens_* = 60), and (3) high score (range 16.50–23.00; *n_children_* = 1092; *n_kindergartens_* = 45). Teachers had the highest score for dose received (satisfaction; 5.79/9 (±2.46)) and fidelity (6.60/11 (±2.91)), and had the lowest scores for dose delivered (1.23/4 (±0.77)). Table 2 shows all the information regarding the different components of the teachers’ process evaluation score. 

For all the food items (yoghurt, cheese, fresh fruit, raw vegetables, aromatized yoghurt, milk-based desserts, chocolate, biscuits, cake, sugar-based desserts, salty snacks, healthy snacks, and unhealthy snacks), no significant interaction effects were found between time and teachers’ process evaluation score (all *p* > 0.003). This shows that there was no change in these food items for preschool children from the control group and preschool children with low, medium, and high teachers’ process evaluation scores between baseline and follow-up.

### 3.4. Association Parents’/Caregivers’ Process Evaluation Score and the Effects on Preschool Children’ Snacking Behavior (Total Sample)

In total, 2792 children (drop-out of 24.14%) had a parents’/caregivers’ process evaluation score. The mean process evaluation score was 8.50 (±4.39) out of a total of 17 (range 0–16). The division of preschoolers based on parents’/caregivers’ process evaluation score was as follows: (1) low score (range 0.00–3.00; *n_children_* = 860), (2) medium score (range 4.00–10.00; *n_children_* = 988), and (3) high score (range 11.00–16.00; *n_children_* = 944). The highest scores were found for dose received—exposure (4.87/10 (±4.07)), and the lowest scores were found for dose received—satisfaction (1.84/6 (±1.59)). Table 3 shows all information regarding the different components of the parents’/caregivers’ process evaluation score.

**Figure 2:**
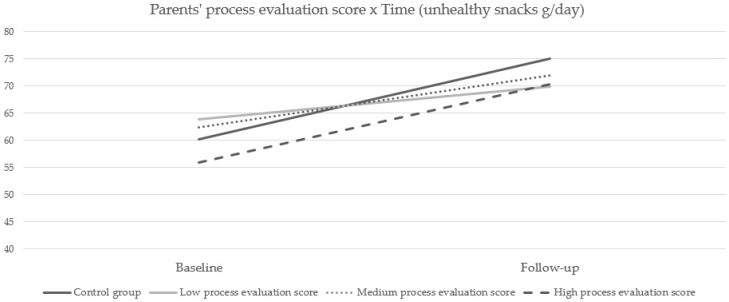
Parents’ process evaluation score for unhealthy snack intake (g/day).

For the intake of unhealthy snacks, a significant interaction effect was found between preschool children from the control group and preschool children with a low parents’/caregivers’ process evaluation score (β = −8.86 (SE = 2.83); *p* = 0.002) going from baseline to follow-up. Preschool children from the control group experienced a larger increase in their daily intake of unhealthy snacks (+14.86 g/day; *p* < 0.001) compared to the preschoolers with a low parents’/caregivers’ process evaluation score, who experienced a smaller increase in unhealthy snack intake (+6.01 g/day; *p* = 0.01). In addition, a significant interaction effect was found between preschool children with a low and preschool children with a high parents’/caregivers’ process evaluation score (β = 8.44 (SE = 3.30); *p* = 0.01) from baseline to follow-up. Preschoolers with a high parents’/caregivers’ process evaluation score had a steeper increase in their daily intake of unhealthy snacks (+14.45 g/day; *p* < 0.001) compared to preschoolers with a low parents’/caregivers’ process evaluation score, who had a smaller increase in their daily intake of unhealthy snacks (+6.01 g/day; *p* = 0.01). No significant differences were found between the other groups (Figure 2).

For yoghurt, cheese, fresh fruit, raw vegetables, aromatized yoghurt, milk-based desserts, chocolate, cake, biscuits, sugar-based desserts, salty snacks, and healthy snacks, no significant effects were found between time and process evaluation score (all *p* > 0.003). This shows that there is no difference between baseline and follow-up in these food items for preschool children from the control group and preschool children with low, medium, and high parents’/caregivers’ process evaluation scores.

## 4. Discussion

The present study targeted two aims. First, the effect of the snacking behavior-module of the ToyBox intervention on European preschool children was investigated in the total sample and in the six different European countries separately (i.e., Belgium, Bulgaria, Germany, Greece, Poland, and Spain). Second, we wanted to put the effect on preschoolers’ snacking behavior into perspective by examining whether a higher teachers’ or parents’/caregivers’ process evaluation score was associated with more positive effects on preschool children’s intake of snacks.

The ToyBox intervention did not cause a change in preschoolers’ daily intake of snacks, since no effects were found in the total sample. The country-specific analyses also showed no intervention effects for the daily snack intake. Surprisingly, every country showed a significant time effect for the intake of chocolate, with an increase in chocolate intake from baseline to follow-up in both the intervention and control group, showing the importance of focusing on snacking behavior in interventions for preschool children, since an apparent increase in chocolate intake was demonstrated over a one-year period. 

The lack of intervention effects are in line with other published papers on the effects of the ToyBox intervention on physical activity [17], beverage consumption [19], and sedentary behavior [18]. Although no effects were found on preschoolers’ snacking behavior itself, the study by Lambrinou et al. (2019) showed that the ToyBox intervention improved the determinants of preschoolers’ snacking behavior [30], which is the first step in changing behaviors. However, it might be possible that not all the determinants of snacking behavior were identified during the PRECEDE (Predisposing, Reinforcing and Enabling Constructs in Educational Diagnosis and Evaluation) phase (embedded within the intervention mapping protocol), which might have led to a lack of effects on preschoolers’ snacking behavior [31]. Other reasons for the lack of effects on the behavior itself can potentially be explained by the duration and implementation intensity of the ToyBox intervention, which were probably too limited to counteract the unfavorable evolution of preschoolers’ snacking behavior. The ToyBox intervention lasted for one school year, with the part focusing on classroom activities taking place for 24 weeks. However, only six out of the 24 weeks focused on healthy snacking, with a one-hour session every week (i.e., 6 h in total). This means that only a limited amount of time was dedicated to the healthy snacking topic. It is suggested that a longer implementation of interventions (i.e., >6 months) might enhance the effects [32]. A recent systematic review on the effectiveness of school-based interventions on four- to 12-year-old children’s physical activity and nutrition, for example, even showed that sustainable interventions, lasting longer than 12 months, were more effective compared to interventions of a shorter duration [33]. Although kindergarten teachers were stimulated to implement the classroom activities for 6 months to increase the odds for having an effective intervention, probably the kindergarten teachers only focused on snacking behavior for a limited amount of time. In addition, one might question whether it is better to focus on a single behavior or to focus on multiple behaviors. The most recent literature suggests that it is better to focus on multiple energy balance-related behaviors (e.g., dietary intake, physical activity, sedentary behavior), since only then do you take the full energy balance into account [33]. However, an important aspect to keep in mind during the implementation of these interventions is that the focus on multiple energy balance-related behaviors should be simultaneous instead of sequential [33]. 

The added value of this paper is the fact that intervention effects on preschoolers’ snacking behavior were put into perspective by combining the effect evaluation with process evaluation. Looking at how well an intervention was implemented and received might provide another interpretation of the effects of the intervention. Other studies have already combined effect and process evaluation and found, for example, that a better implementation and thus a higher process evaluation score prevented unfavorable changes in certain behaviors (e.g., physical activity, sedentary behavior, fruit and vegetable intake) compared to lower process evaluation scores [12,13]. 

Within the ToyBox study, no effects of a higher process evaluation score in teachers were found for snacking behavior. This means that the results that were found for physical activity, sedentary behavior, and beverage consumption [17,18,19] were unfortunately not replicated for snacking behavior. In general, also no different effects on preschoolers’ snacking behavior were found according to parents’/caregivers’ process evaluation score. Only one significant effect was found, which was not as expected, since preschoolers with parents/caregivers with a low process evaluation score showed a smaller increase in their intake of unhealthy snacks from baseline to follow-up, compared to a steep increase for preschoolers with parents/caregivers with a high process evaluation score and the control group. It must be noted that preschoolers’ parents/caregivers were only passively involved in the ToyBox intervention with the use of newsletters and tip cards. It might be that, because of only passively involving parents, the effect on preschool children’s snacking behavior might be limited to the minimum. 

It must be noted that, for teachers and parents/caregivers, low overall process evaluation scores were found. The mean process evaluation score for teachers was only 14.58/24, and the variation within the three levels (i.e., low, medium, and high) was very small, which might be the reason for the absence of additional and stronger effects. The fact that we generally found low process evaluation scores in teachers could be due to the fact that the intervention was not developed together with the teachers and a top-down approach was used. This means that the researchers decided upon the content, duration, frequency, and mode of delivery. Before the development of the intervention, teachers other than the ones involved in the intervention itself participated in focus groups and then researchers used the information from the focus groups to develop the intervention. However, the content and specifics of the intervention were not checked against the expertise of the teachers involved in the focus groups, although it is important to mention that the intervention materials used in the ToyBox intervention were improved for future use based on feedback from kindergarten teachers and parents/caregivers. A future strategy might be to use an approach that employs participatory action research [34], which means that the target group (in this case, preschool children) and implementers (in this case, parents and teachers) are actively involved in developing, implementing, and evaluating the intervention (i.e., a bottom-up approach). Kindergarten teachers are important role models in the life of preschool children, and the results from focus groups with kindergarten teachers that were conducted within the ToyBox study also made it clear that kindergarten teachers perceive themselves as important role models [24]. However, kindergarten teachers also need to understand that they have an influence on the quality of intervention implementation as well. Therefore, future intervention studies should more thoroughly include the determinants of implementation specifically for implementers while designing an intervention. This might induce kindergarten teachers to be more engaged in implementing the intervention as intended, and this might heighten the process evaluation scores and eventually lead to larger effects. In addition, it might be questioned as to what extent teachers reliably filled in the monthly logbooks, since teachers from the focus groups mentioned that they did not want to have any additional workload [25]. Teachers’ logbooks had to be filled in on a monthly basis, and it might be that teachers did not fill them in conscientiously or that they provided socially desirable answers. Additionally, the fact that snacking behavior was the third behavior in line that teachers had to focus on might be a reason for teachers to have less thoroughly implemented the intervention content. 

The low parental process evaluation score (8.50/17) was much lower than the one aimed for, although parents/caregivers were only passively involved. Highest scores were found for dose delivered, which means that parents/caregivers received the newsletters, tip cards, and posters and that some of them read the materials. However, low scores for satisfaction were found (dose received: 1.84/6). which might mean that parents/caregivers were not satisfied with the practicality, design, and large amount of text in the materials. Thus, passively involving parents/caregivers via written materials delivered at home with recommendations and motivational messages on how to decrease preschoolers’ intake of unhealthy snacks might not be sufficient to adequately change parental determinants of preschool children’s snacking behavior. A more promising strategy might be to actively and intensively involve parents/caregivers in future interventions (e.g., sessions for parents, parent–child workshops). Additionally, similar to what we have suggested for kindergarten teachers, involving parents/caregivers through a participatory approach to develop an active parental intervention module to target preschoolers’ snacking behavior might be of added value [34]. 

This paper is the last one of the ToyBox papers looking at the effect of the ToyBox intervention on preschoolers’ energy balance-related behaviors in combination with process evaluation. The results of the present study should be considered in the light of its limitations and strengths. One might question whether the general lack of effects could have been predicted beforehand. Comparable with the European IDEFICS study, some methodological issues and weaknesses were present in the ToyBox study, which include the short intervention duration, as well as the fact that some intervention components were actually not delivered as intended [35]. We might question whether a one-size-fits-all intervention is the optimal way forward, since the determinants of behaviors are complex, different, and unique across cultures and countries [35]. A standardized intervention such as the ToyBox intervention, although there is room for limited cultural and local adaptations, might be useful for evaluation purposes, but the effectiveness of such interventions may be amplified when a participatory approach and more extensive local adaptations are allowed. Even though randomized controlled trials are the gold standard in medical research [36], their relevance in health promotion research might be questioned [37,38]. Therefore, Hawe et al. (2004) suggested taking another approach by looking at the components of an intervention (e.g., the content of the classroom activity guides within the ToyBox intervention) as the “mechanisms” in the change process rather than standardizing these components [39]. This means that these components can take on diverse forms as per the local and individual context [39]. Therefore, future intervention studies might take the approach of Hawe et al. (2004) to increase the effectiveness of interventions. The ToyBox study is currently scaling up on a global basis following this approach. 

Another limitation of the current study is that the data used to compute the process evaluation scores were self-reported, and not all teachers filled in all logbooks. This might have induced bias. Furthermore, the calculation of the process evaluation scores is not standardized. The method used in the current study was based on previous studies [12,17,18,19] but still had some limitations, since not all components of the process evaluation framework of Saunders et al. (2005) [11] could be used to compute the process evaluation scores for the teachers and parents/caregivers. Moreover, it might also be relevant to check whether higher scores on the key elements induce larger intervention effects. Therefore, the process evaluation scores provided in the current study give a general picture of the level of teachers’ and parents/caregivers’ process. Future research should invest in developing a standardized means to combine the effect and process evaluation of interventions, and could use a combination of quantitative methods (e.g., Likert-type scale questions) to calculate a quantitative process evaluation score which can be linked to effect evaluation, as well as qualitative methods (e.g., open-ended questions, semi-structured face-to-face interviews) to explore the variety of experiences with the intervention and to get more in-depth information that cannot be captured by items in a questionnaire. 

The strengths of the current study are the large sample of preschoolers from six European countries and the use of a cluster randomized controlled trial with a baseline and follow-up measurement. In addition, the use of process evaluation questionnaires for both teachers and parents/caregivers is also a strength, together with the use of the theory-based process evaluation model of Saunders et al. [11] to compose the process evaluation questions. Moreover, all the countries followed standardized protocols, materials, and methods for the implementation and evaluation (effect and process) of the ToyBox intervention.

## 5. Conclusions

The ToyBox intervention showed no effect on European preschoolers’ snacking behavior. In general, there were also no differences in effect on preschoolers’ snack intake when looking at the process evaluation scores of the kindergarten teachers and preschoolers’ parents/caregivers. One of the possible reasons for this might be the limited duration and dose, in combination with the fact that there was a focus on multiple behaviors. To induce larger effects, a less standardized intervention which is tailored to local needs might be needed.

## Figures and Tables

**Table 1:** Overview of the process evaluation questions to calculate the process evaluation score (score on 24) for teachers.

Questionnaire Used	Fidelity = Whether the Intervention Was Implemented as Planned by the Teachers and Received by the Children	Dose Delivered = How Much Was Delivered by the Teachers and Received by the Children	Dose Received—Satisfaction = How the Intervention Was Received by the Teachers
Questionnaire Teachers’ physical activity logbook (first focus)	*“When did you deliver the 1st snacking newsletter to the parents? Not delivered – in week 9 – in week 10 – in week 11 – in week 12”* 1 = yes (in weeks 9, 10, 11 or 12) 0 = not delivered *“When did you deliver the 1st snacking tip-card to the parents? Not delivered – in week 9 – in week 10 – in week 11 – in week 12”* 1 = yes (in weeks 9, 10, 11 or 12) 0 = not delivered *“When did you deliver the snacking poster to the parents? Not delivered – in week 9 – in week 10 – in week 11 – in week 12”* 1 = yes (in weeks 9, 10, 11 or 12) 0 = not delivered *“All planned activities were performed. Totally disagree – disagree – neither disagree nor agree – agree – totally agree”* 1 = score ≥ mean value of 3.46 0 = score < mean value of 3.46 *“Did you implement the classroom activities as described in the manual for snacking? Never – rarely – sometimes – often – always”* 1 = score ≥ mean value of 2.37 0 = score < mean value of 2.37 *“Was the Magic Snack Plate available at scheduled times? Never – rarely – sometimes – often – always”* 1 = score ≥ mean value of 2.22 0 = score < mean value of 2.22	*“Did you devote on average at least one hour per week in the classroom activities as described in the manual? Never – rarely – sometimes – often – always”* 1 = score ≥ mean value of 2.06 0 = score < mean value of 2.06 Sum score of 27 items related to classroom activities for snacking behavior (implementation of 10 kangaroo stories, 8 sensory perception games, 2 experiments and 7 excursions; answer possibilities: yes - no) (mean score: 6.68) 1 = score ≥ mean value of 6.68 0 = score < mean value of 6.68	*“It was easy to read and understand the text in the Classroom Activity Guide for snacking”* 1 = score ≥ mean value of 4.11 0 = score < mean value of 4.11 *“The amount of information and activities in the Classroom Activity Guide for snacking were appropriate”* 1 = score ≥ mean value of 3.92 0 = score < mean value of 3.92 *“It was easy to implement the activities described in the Classroom Activity Guide for snacking”* 1 = score ≥ mean value of 3.65 0 = score < mean value of 3.65 *“I enjoyed the activities I delivered this month”* 1 = score ≥ mean value of 3.96 0 = score < mean value of 3.96 *“The activities I delivered this month were enjoyed by the children”* 1 = score ≥ mean value of 4.14 0 = score < mean value of 4.14 *“The information presented in the Classroom Activity Guide for snacking, the content of the material and the way the activities should be delivered are appropriate to achieve the goals”* 1 = score ≥ mean value of 3.86 0 = score < mean value of 3.86
Questionnaire Teachers’ physical activity logbook (repetition period)	*“When did you deliver the 2nd physical snacking newsletter to the parents? Not delivered – in week 21 – in week 22”* 1 = yes (in weeks 21 or 22) 0 = not delivered *“When did you deliver the 2nd snacking tip-card to the parents? Not delivered – in week 21 – in week 22”* 1 = yes (in weeks 21 or 22) 0 = not delivered *“All planned activities were performed. Totally disagree – disagree – neither disagree nor agree – agree – totally agree”* 1 = score ≥ mean value of 3.40 0 = score < mean value of 3.40 *“Did you implement the classroom activities as described in the manual for snacking? Never – rarely – sometimes – often – always”* 1 = score ≥ mean value of 2.52 0 = score < mean value of 2.52 *“Was the Magic Snack Plate available at scheduled times? Never – rarely – sometimes – often – always”* 1 = score ≥ mean value of 2.29 0 = score < mean value of 2.29	*“Did you devote on average at least one hour per week in the classroom activities as described in the manual? Never – rarely – sometimes – often – always”* 1 = score ≥ mean value of 2.21 0 = score < mean value of 2.21 Sum score of 27 items related to classroom activities for snacking behavior (implementation of 10 kangaroo stories, 8 sensory perception games, 2 experiments, and 7 excursions; answer possibilities: yes/no) (mean score: 7.11) 1 = score ≥ mean value of 7.11 0 = score < mean value of 7.11	*“It was easy to implement the activities described in the Classroom Activity Guide for snacking”* 1 = score ≥ mean value of 3.70 0 = score < mean value of 3.70 *“I enjoyed the activities I delivered this month”* 1 = score ≥ mean value of 4.02 0 = score < mean value of 4.02 *“The activities I delivered this month were enjoyed by the children”* 1 = score ≥ mean value of 4.15 0 = score < mean value of 4.15
Mean score (± standard deviation)/ maximum score	6.60 (±2.91)/11	1.23 (±0.77)/4	5.79 (±2.46)/9

**Table 2:** Overview process evaluation questions to calculate the process evaluation score (score on 17) for parents.

Dose Delivered	Dose Received—Exposure	Dose Received—Satisfaction
*“Did you or your partner receive the materials regarding physical activity?”* (one score for each component: Newsletter 1, Newsletter 2, Tip card 1, Tip card 2, Poster) 1 = yes 0 = no and I do not know *“Did you or your partner read the materials regarding physical activity?”* (one score for each component: Newsletter 1, Newsletter 2, Tip card 1, Tip card 2, Poster) 1 = yes 0 = no and I do not know	*“Did you implement the suggested activities of the ToyBox Newsletters and Tip-cards? Never – rarely – sometimes – often – always”* 1 = score ≥ mean value of 3.07 0 = score < mean value of 3.07	*“In general, how easy was it to understand the text in the ToyBox Newsletters and Tip Cards? Very difficult – difficult – easy – very easy”* 1 = score ≥ mean value of 3.45 0 = score < mean value of 3.45 *“In general, did you find the information provided in the ToyBox Newsletters and Tip Cards trustful? Not at all – to a little degree – neither trustful or not trustful – to some degree – to a large degree”* 1 = score ≥ mean value of 4.39 0 = score < mean value of 4.39 *In general, how useful did you find the Suggestions and Tips for parents in the ToyBox Newsletters and Tip Cards? Not useful at all – a little useful – somewhat useful – very useful”* 1 = score ≥ mean value of 3.25 0 = score < mean value of 3.25 *Did you/ your partner and your child enjoy the ToyBox activities conducted with the family? I did not enjoy it at all – I did not enjoy it so much – I enjoyed it – I enjoyed it a lot”* 1 = score ≥ mean value of 2.87 0 = score < mean value of 2.87 *“In general, what did you think about the amount of text in the ToyBox Newsletters and Tip cards? Far too much – too much – about right – too little – far too little”* 1 = 3 0 = > 3 and < 3 *“In general, what did you think of the design (colours, lay out, type of letters) of the ToyBox Newsletters and Tip Cards? I did not like it at all – I did not like it so much – I liked it – I liked it a lot”* 1 = score ≥ mean value of 3.08 0 = score < mean value of 3.08
4.87 (±4.07)/10 *	0.33 (±0.47)/1 *	1.84 (±1.59)/6 *

* Mean score (± standard deviation)/maximum score.

**Table 3:** Time and interaction effects for the different food categories in the total sample (adjusted for age and sex).

*n* = 4970 (I = 3255; C = 1715)		PRE (g/day)	POST (g/day)	Time	Time * Condition
				Change	ß
Plain yoghurt	I	39.71	37.69	−2.49	0.47
	C	40.72	38.23		
Cheese	I	11.86	11.69	−0.44	0.27
	C	11.91	11.48		
Fresh fruit	I	130.68	128.19	−4.08 *	1.58
	C	129.49	125.41		
Raw vegetables	I	54.46	56.42	1.60	0.36
	C	56.37	57.97		
Sugared or aromatized yoghurt	I	42.19	40.02	−4.97 *	2.79
	C	42.18	37.21		
Chocolate and candy bars	I	11.11	27.94	15.60 *	1.23
	C	11.04	26.64		
Milk-based desserts	I	20.71	18.06	−3.49 *	0.84
	C	20.39	16.90		
Cakes	I	10.57	10.98	0.23	0.18
	C	10.23	10.46		
Biscuits	I	12.30	12.24	−0.90 *	0.84
	C	11.59	10.69		
Sugar-based desserts	I	2.00	2.33	0.55 *	−0.22
	C	2.21	2.76		
Salty snacks	I	5.13	5.09	−0.14	0.10
	C	5.22	5.08		
Healthy snacks	I	228.58	223.82	−0.70	4.06
	C	229.55	228.85		
Unhealthy snacks	I	60.89	75.22	11.02 *	3.31
	C	59.71	70.73		

* *p* < 0.003; I = intervention group; C = control group.

**Table 4:** Time and interaction effects for the different food categories for the six intervention countries separately (adjusted for age and sex).

Food Categories	BELGIUM *n* = 771 (I = 468; C = 303)					BULGARIA *n* = 644 (I = 458; C = 186)					GERMANY *n* = 882 (I = 550; C = 332)				
		PRE (g/day)	POST (g/day)	Time	Time * Condition		PRE (g/day)	POST (g/day)	Time	Time * Condition		PRE (g/day)	POST (g/day)	Time	Time * Condition
				Change	ß				Change	ß				Change	ß
Plain yoghurt	I	20.87	19.79	1.10	−2.18	I	71.54	64.77	−10.08	3.31	I	17.12	18.78	2.20	−0.60
	C	17.23	18.32			C	75.76	65.68			C	17.27	19.48		
Cheese	I	8.13	7.59	−0.10	−0.44	I	14.22	13.54	−0.56	−0.12	I	10.57	10.71	0.02	0.11
	C	8.19	8.08			C	14.54	13.97			C	10.02	10.04		
Fresh fruit	I	129.54	137.79	4.40	3.85	I	150.22	136.06	0.51	−14.67	I	135.66	136.13	−4.52	4.99
	C	135.95	140.36			C	146.83	147.34			C	139.74	135.22		
Raw vegetables	I	31.17	40.48	2.09	7.21	I	101.32	99.50	4.89	−6.71	I	77.53	77.78	−2.09	2.35
	C	36.04	38.13			C	101.23	106.12			C	84.21	82.12		
Sugared or aromatized yoghurt	I	41.59	39.98	−3.61	2.00	I	19.73	17.76	−0.57	−1.40	I	46.51	43.55	0.36	−3.33
	C	46.55	42.94			C	18.00	17.43			C	46.56	46.91		
Chocolate and candy bars	I	5.37	17.13	11.65 *	0.11	I	15.19	30.06	20.55 *	−5.68	I	13.63	37.83	27.36 *	−3.16
	C	5.17	16.82			C	12.94	33.49			C	14.16	41.52		
Milk-based desserts	I	22.82	21.56	−1.19	−0.07	I	19.88	17.85	−1.50	−0.54	I	20.66	17.75	−4.01	1.09
	C	25.81	24.62			C	18.30	16.81			C	20.83	16.82		
Cakes	I	19.52	16.25	−1.99	−1.29	I	9.56	9.84	−0.23	0.51	I	9.04	10.33	2.01	−0.72
	C	19.06	17.07			C	9.89	9.66			C	8.46	11.56		
Biscuits	I	18.29	16.76	0.57	−2.11	I	10.28	9.28	−0.11	−0.88	I	6.15	5.56	−0.45	−0.15
	C	17.88	18.45			C	9.72	9.61			C	5.95	5.50		
Sugar-based desserts	I	2.85	2.89	−0.20	0.24	I	1.67	2.54	−0.16	1.03 *	I	3.12	3.84	0.27	0.45
	C	3.09	2.89			C	2.36	2.20			C	3.62	3.89		
Salty snacks	I	3.57	3.80	0.54	−0.31	I	9.00	8.48	−1.06	0.54	I	3.06	3.21	0.29	−0.14
	C	4.10	4.63			C	9.37	8.31			C	2.53	2.82		
Healthy snacks	I	182.65	198.77	10.54 *	−5.59	I	324.79	308.99	−7.70	7.58	I	263.68	266.05	−0.98	−4.34
	C	187.59	198.13			C	314.79	307.09			C	270.90	269.22		
Unhealthy snacks	I	69.62	75.62	10.82	−4.82	I	63.51	76.37	18.06 *	−5.19	I	55.40	76.15	25.11 *	−4.37
	C	71.61	82.43			C	61.25	79.31			C	53.94	79.05		
**Food Categories**	**GREECE** ***n* = 825 (I = 612; C = 213)**					**POLAND** ***n* = 1,021 (I = 637; C = 384)**					**SPAIN** ***n* = 827 (I = 530; C = 297)**				
		**PRE** **(g/day)**	**POST** **(g/day)**	**Time**	Time * Condition		**PRE** **(g/day)**	**POST** **(g/day)**	**Time**	Time * Condition		**PRE** **(g/day)**	**POST** **(g/day)**	**Time**	Time * Condition
				**Change**	**ß**				**Change **	**ß**				**Change**	**ß **
Plain yoghurt	I	34.52	34.07	−2.94	2.49	I	26.24	24.02	−0.57	−1.65	I	51.74	44.82	−6.79	−0.13
	C	42.79	39.85			C	24.96	24.39			C	52.38	45.59		
Cheese	I	19.83	20.40	0.09	−0.21	I	10.46	9.95	−0.29	−0.22	I	9.86	8.85	−0.23	−0.78
	C	20.61	20.70			C	10.76	10.47			C	9.92	9.69		
Fresh fruit	I	132.31	125.31	−6.01	−0.98	I	130.92	128.03	−1.48	−1.42	I	106.82	106.82	−7.41	−1.85
	C	132.56	126.55			C	126.33	124.85			C	108.04	100.63		
Raw vegetables	I	66.74	62.27	5.06	−8.98	I	59.84	63.77	2.77	1.51	I	24.62	26.79	1.78	0.39
	C	64.94	70.00			C	62.59	65.36			C	22.81	24.59		
Sugared or aromatized yoghurt	I	30.13	24.30	−2.23	−3.60	I	49.38	41.08	−3.38	−4.92	I	54.61	42.28	−3.09	−4.24
	C	30.62	28.38			C	44.02	40.65			C	56.28	53.19		
Chocolate and candy bars	I	16.31	31.34	14.17 *	0.86	I	11.61	24.77	13.56 *	−0.40	I	9.00	23.26	14.16 *	0.10
	C	17.66	31.83			C	10.37	23.93			C	9.12	23.28		
Milk-based desserts	I	13.66	6.70	−5.16	2.74	I	30.96	25.61	−4.48	−0.88	I	22.07	16.00	0.83	−6.90 *
	C	9.71	4.54			C	29.20	24.73			C	20.79	19.96		
Cakes	I	11.37	13.53	−0.28	2.44	I	15.92	15.35	1.45	−2.02	I	4.90	5.76	0.54	0.32
	C	12.22	11.94			C	13.99	15.43			C	5.05	5.58		
Biscuits	I	10.94	10.19	0.73	−1.48	I	5.85	5.38	−0.90	0.43	I	18.61	17.32	0.26	−1.55
	C	9.67	10.40			C	5.56	4.66			C	17.15	17.41		
Sugar-based desserts	I	1.26	1.75	0.24	0.25	I	2.92	3.66	1.00	−0.26	I	1.22	1.63	0.40	0.01
	C	1.10	1.34			C	2.96	3.96			C	1.09	1.49		
Salty snacks	I	5.01	4.99	0.34	−0.36	I	3.17	3.71	0.21	0.33	I	6.47	5.11	−0.96	−0.41
	C	4.77	5.11			C	3.62	3.83			C	7.05	6.10		
Healthy snacks	I	242.96	231.59	2.50	13.87	I	222.11	218.42	8.23	4.50	I	196.29	180.79	−10.54 *	4.96
	C	247.23	249.73			C	218.40	226.63			C	187.49	176.95		
Unhealthy snacks	I	51.84	66.05	10.95	3.26	I	69.77	76.31	8.36	−1.82	I	61.36	67.03	13.83 *	−8.16
	C	53.02	63.97			C	64.77	73.13			C	60.13	73.95		

* *p* < 0.003; I = intervention group; C = control group

## Supplementary Materials

The following are available online at https://www.mdpi.com/1660-4601/17/19/7312/s1. Supplementary file 1: Questions and answer possibilities on pre-schoolers’ snack consumption; Supplementary file 2: CONSORT checklist.
