# Supplementary material for: Combining Effect and Process Evaluation on European Preschool Children’s Snacking Behavior in a Kindergarten-Based, Family-Involved Cluster Randomized Controlled Trial: The ToyBox Study
Source: Int J Environ Res Public Health. 2020 Oct 7;17(19):7312. doi: 10.3390/ijerph17197312 (PMC7579655; doi:10.3390/ijerph17197312)
Supplement: Supplementary file 1 [file ijerph-17-07312-s001.zip › Supplementary Table 1.docx]

| **Supplementary Table 1.** Questions and answer possibilities on pre-schoolers’ snack consumption | | |
| --- | --- | --- |
|  | How often does your child consume the following products? | What is the average amount per day? |
| Plain yoghurt (without added sugar)  *Healthy snack* | 1. Never or less than once per month  2. 1-3 days per month  3. 1 day per week  4. 2-4 days per week  5. 5-6 days per week  6. Every day | 1. 65g or less  2. Between 65 and 95g  3. 195g or more |
| Sugared or aromatised yoghurt  *Unhealthy snack* | 1. Never or less than once per month  2. 1-3 days per month  3. 1 day per week  4. 2-4 days per week  5. 5-6 days per week  6. Every day | 1. 65g or less  2. Between 65g and 95g  3. 195g or more |
| Cheese  *Healthy snack* | 1. Never or less than once per month  2. 1-3 days per month  3. 1 day per week  4. 2-4 days per week  5. 5-6 days per week  6. Every day | 1. 10g or less  2. Between 10 and 20g  3. Between 20 and 30g  4. Between 30 and 40g  5. Between 40 and 50g  6. 50g or more |
| Fresh fruit  *Healthy snack* | 1. Never or less than once per month  2. 1-3 days per month  3. 1 day per week  4. 2-4 days per week  5. 5-6 days per week  6. Every day | 1. 30g or less  2. Between 30 and 60g  3. Between 60 and 90g  4. Between 90 and 120g  5. Between 120 and 150g  6. Between 150 and 180g  7. Between 180 and 210g  8. Between 210 and 240g  9. Between 240 and 270g  10. 270g or more |
| Raw vegetables  *Healthy snack* | 1. Never or less than once per month  2. 1-3 days per month  3. 1 day per week  4. 2-4 days per week  5. 5-6 days per week  6. Every day | 1. 30g or less  2. Between 30 and 60g  3. Between 60 and 90g  4. Between 90 and 120g  5. Between 120 and 150g  6. Between 150 and 180g  7. Between 180 and 210g  8. Between 210 and 240g  9. Between 240 and 270g  10. 270g or more |
| Milk-based desserts  *Unhealthy snack* | 1. Never or less than once per month  2. 1-3 days per month  3. 1 day per week  4. 2-4 days per week  5. 5-6 days per week  6. Every day | 1. 50g or less  2. Between 50 and 100g  3. Between 100 and 150g  4. Between 150 and 200g  5. 200g or more |
| Chocolate and  candy bars  *Unhealthy snack* | 1. Never or less than once per month  2. 1-3 days per month  3. 1 day per week  4. 2-4 days per week  5. 5-6 days per week  6. Every day | 1. 25g or less  2. Between 25 and 50g  3. Between 50 and 75g  4. Between 75 and 100g  5. Between 100 and 125g  6.125g or more |
| Sugar-based desserts  *Unhealthy snack* | 1. Never or less than once per month  2. 1-3 days per month  3. 1 day per week  4. 2-4 days per week  5. 5-6 days per week  6. Every day | 1. 5g or less  2. Between 5 and 10g  3. Between 10 and 15g  4. Between 15 and 20g  5. Between 20 and 25g  6. Between 25 and 30g  7. Between 30 and 35g  8. 35g or more |
| Cakes  *Unhealthy snack* | 1. Never or less than once per month  2. 1-3 days per month  3. 1 day per week  4. 2-4 days per week  5. 5-6 days per week  6. Every day | 1. 35g or less  2. Between 35 and 70g  3. Between 70 and 105g  4. Between 105 and 140g  5. Between 140 and 175g  6. Between 175 and 210g  7. Between 210 and 245g  8. 245g or more |
| Biscuits  *Unhealthy snack* | 1. Never or less than once per month  2. 1-3 days per month  3. 1 day per week  4. 2-4 days per week  5. 5-6 days per week  6. Every day | 1. 15g or less  2. Between 15 and 30g  3. Between 30 and 45g  4. Between 45 and 60g  5. 60g or more |
| Salty snacks  *Unhealthy snack* | 1. Never or less than once per month  2. 1-3 days per month  3. 1 day per week  4. 2-4 days per week  5. 5-6 days per week  6. Every day | 1. 25g or less  2. Between 25 and 75g  3. 75g or more |
